# Targeted Next-Generation Sequencing Identifies Actionable Targets in Estrogen Receptor Positive and Estrogen Receptor Negative Endometriod Endometrial Cancer

**DOI:** 10.3389/fphar.2018.00750

**Published:** 2018-07-13

**Authors:** Siti Syazani Suhaimi, Nurul-Syakima Ab Mutalib, Sheau S. Khor, Reena Rahayu Md Zain, Saiful Effendi Syafruddin, Nadiah Abu, Ahmad Zailani Hatta Mohd Dali, Rahman Jamal

**Affiliations:** ^1^UKM Medical Molecular Biology Institute, Universiti Kebangsaan Malaysia, UKM Medical Center, Kuala Lumpur, Malaysia; ^2^Thermo Fisher Scientific, Shah Alam, Malaysia; ^3^Department of Pathology, Faculty of Medicine, Universiti Kebangsaan Malaysia, Kuala Lumpur, Malaysia; ^4^Department of Obstetrics and Gynaecology, Faculty of Medicine, Universiti Kebangsaan Malaysia, Kuala Lumpur, Malaysia

**Keywords:** endometrial cancer, endometrioid subtype, next-generation sequencing, estrogen receptor, *WHSC1*, Fulvestrant

## Abstract

Endometrioid endometrial cancer (EEC) is the commonest form of endometrial cancer and can be divided into estrogen receptor (ER) positive and negative subtypes. The mutational profiles of EEC have been shown to aid in tailoring treatment; however, little is known about the differences between the gene mutation profiles between these two subtypes. This study aims to investigate the gene mutation profile in ER positive and negative EEC, and to further elucidate the role of *WHSC1* mutations in this cancer. EEC and normal endometrial tissues were obtained from 29 patients and subjected to next-generation sequencing (NGS) using Ion Ampliseq Comprehensive Cancer Panel^TM^ targeting 409 cancer related. A total of 741 non-synonymous alterations were identified from 272 genes in ER positive subtype while 448 non-synonymous variants were identified from 221 genes in ER negative subtype. PTEN is the most frequently altered gene in ER positive subtype (64%, 7/11) while ARID1A is the most frequently altered gene in ER negative subtype (50%, 4/8). We also identified alterations in ERRB3 (36%, 4/11), GNAS (36%, 4/11), and WHSC1 (27%, 3/11) in the ER positive subtype. *WHSC1* R1126H and L1268P were shown to significantly increase cell viability, proliferation, migration, and survival. In addition, reduction in ER expression sensitized EEC-1 cell with *WHSC1* L1268P mutant to Fulvestrant treatment. We revealed the mutational spectra of ER positive and ER negative EEC that could lead to better understanding of the biological mechanisms of endometrial cancer and may ultimately result in improvement of treatment options and patient prognosis.

## Introduction

Endometrial cancer is the sixth most common cancer diagnosed among women with approximately 320,000 new cases and 76,000 deaths worldwide each year (Ferlay et al., 2013). Early detection is common as the disease is symptomatic even at an early stage and therefore is often diagnosed at Stage I (Morice et al., 2016). Despite the fact that most cases are diagnosed early, the incidence and mortality rates for endometrial cancer have been rising in both developed as well as developing countries and are expected to rise further with the increasing aging population and high prevalence of obesity (Ferlay et al., 2015; Morice et al., 2016). Furthermore, although endometrial cancer is generally thought to be a cancer of the postmenopausal women, 14% of cases are diagnosed during premenopausal period with 5% of the patients younger than 40-year old (Duska et al., 2001).

In the past three decades, endometrial cancer has been broadly divided into two subtypes based on histological characteristics, expression of estrogen receptor (ER) and grade (Bokhman, 1983). Majority of endometrial cancer which are designated as Type I endometrioid endometrial cancer (EEC) account for more than 75% of all cases (Carlson et al., 2012), follow the estrogen-related pathway, and arise from hyperplastic endometrium background (Bansal et al., 2009). Frequently diagnosed in premenopausal and young postmenopausal women, Type I EEC is often low grade and well-differentiated thus carrying a favorable outcome (Garg and Soslow, 2014). The less common Type II non-endometrioid endometrial cancer (NEEC), which accounts for 10–20% of all cases, follows the estrogen unrelated pathway and arise in background of atrophic endometrium (Doll et al., 2008). This type has a poorer prognosis and usually presents at the advanced stage, especially in older postmenopausal women (Amant et al., 2005). NEEC is also associated with a high mortality, reduced survival rates, and tendency to recur. This dualistic categorization has been incorporated into clinical decision-making algorithms to define high-risk patients, yet its prognostic value remains limited because one-fifth of EEC (i.e., Type I) will eventually relapse, whereas half of NEEC (i.e., Type II) do not (Bokhman, 1983). Furthermore, 15–20% of EEC are high-grade lesions, and it is unclear where they fit into the dualistic model (Zannoni et al., 2013).

This dualistic model has also been supported by molecular studies where Type I EEC has been symbolized by frequent alterations in *PTEN*, *PIK3CA*, *KRAS*, *CTNNB1*, and *ARID1A* as well as defects in DNA mismatch repair (MMR) resulting in the microsatellite instability (MSI) phenotype (O’Hara and Bell, 2012). In contrast, mutations in *TP53* and *PP2R1*A as well as a high expression of oncogene *Her2/ERBB2* are the major genetic changes among Type II NEEC (Lax et al., 2000; O’Hara and Bell, 2012). However, this classification is controversial due to existence of a minority of endometrial cancer cases with overlapping clinical features, morphological and molecular characterization which represents a major obstacle to effective cancer treatment (Talhouk and McAlpine, 2016). For example, Type I EEC is not completely ER positive and loss of ER expression is correlated with aggressive behavior, high grade histology, and poor survival rate (Maniketh et al., 2014; Backes et al., 2016). In contrast to breast cancer, where the ER status (in addition to progesterone) is a pillar for its molecular and clinicopathological classification (Nadji et al., 2005), a comprehensive view of the mutation spectrum between ER positive and ER negative in the same molecular subtype (i.e., endometrioid) has not been fully elucidated. Therefore, the aim of this study is to characterize somatic gene alterations in ER positive and ER negative EEC using targeted deep sequencing of 409 cancer-related genes.

## Materials and Methods

### Clinical Specimen, DNA Extraction, and Quality Assessment

Fresh frozen tissues of EECs (*n* = 19) were obtained after hysterectomy from the patients admitted to the Universiti Kebangsaan Malaysia Medical Centre (UKMMC). The cancers were classified according to the World Health Organization (WHO) classification of tumors of the female reproductive system (Pecorelli, 2009). In addition, fresh frozen normal endometrium (*n* = 10) were obtained from patients surgically treated for non-malignant endometrial diseases. This study was approved by the Universiti Kebangsaan Malaysia Research Ethics Committee (UKM 1.5.3.5/244/AP-2012-011) and carried out in accordance with the approved guidelines. All patients provided written informed consent for their tissue samples to be used for research. Immunohistochemical staining for ER (antibody clone 1D5, DAKO, Carpinteria, CA, United States) and Hematoxylin and Eosin (H&E) staining was performed on the frozen sections and then reviewed by a pathologist. Immunohistochemical staining for ER was scored as positive if 1% or more of tumor nuclei were immunoreactive and negative if less than 1% of tumor cell nuclei were immunoreactive. Only cancer specimens containing more than 80% cancer cells and normal tissues with less than 20% necrosis were subjected to DNA isolation using the QIAamp^®^ DNA Mini Kit (Qiagen, Valencia, CA, United States) following the manufacturer’s protocol.

### Next-Generation Sequencing

The Ion Ampliseq^TM^ Comprehensive Cancer Panel V2 (Life Technologies, Guilford, CT, United States), which covers 409 oncogenes and tumor suppressor genes, was used for library preparation (**Supplementary Table S1**) according to manufacturer’s instruction. The libraries were then normalized to 12–25 pM for template preparation on the Ion One Touch (Life Technologies, Guilford, CT, United States). The clonal amplification of the DNA libraries on the Ion Sphere Particles (ISPs) was carried out using emulsion PCR and the subsequent isolation of templated ISPs was performed using Ion OneTouch ES (Life Technologies, Guilford, CT, United States). Subsequently, next-generation sequencing (NGS) was performed on the Ion Torrent Personal Genome Machine (PGM^TM^) using 318^TM^ chip and Ion Torrent PGM Sequencing 200 kit V2 (Life Technologies, Guilford, CT, United States).

### Bioinformatics Analyses

#### Read Mapping and Variant Calling

Data from the sequencing runs were automatically transferred to the Torrent Server hosting the Torrent Suite Software v4.0.3. The readings were mapped to the reference genome (hg19) using the Torrent Mapping Alignment program (TMAP). Variant calling was generated using Torrent Variant Caller v 4.0 with low stringency settings (Life Technologies, Guilford, CT, United States).

#### Variant Annotation and Prioritization

The functional effects of the variants were further annotated using ANNOVAR (Wang et al., 2010) with respect to gene regions and filter-based annotations. Prediction on protein impact of variants was performed using SIFT, PolyPhen2 HDIV, PolyPhen2 HVAR, LRT, MutationTaster, MutationAssessor, FATHMM, GERP++, PhyloP, and SiPhy databases that are available in ANNOVAR. We classified SNVs as pathogenic if they were observed to be deleterious by three or more than three SNV protein prediction algorithms.

To evaluate which mutations could be actionable and to prioritize for the true somatic mutations, several additional filtering steps were performed. Details on filtrations steps are illustrated in **Supplementary Figure S1**. The alignment of each candidate variant was manually inspected to check for sequencing artifacts and alignment errors using the Integrated Genomic Viewer (IGV) (Thorvaldsdóttir et al., 2013). Oncoprint diagram and lollipop plot were created using Oncoprinter and Mutation Mapper tools, respectively (Cerami et al., 2012; Gao et al., 2013). The detected mutations were compared to those in available cancer databases from the COSMIC v82 (Forbes et al., 2017), MyCancerGenome (Van Allen et al., 2013), and International Cancer Genome Consortium (ICGC) database (Zhang et al., 2011). Cancer Genome Interpreter was used to classify driver mutation among the somatic alterations (Tamborero et al., 2018).

To identify relevant genes for further characterization, we prioritized the genes involved in endometrial cancer carcinogenesis using following strategies: (1) genes associated with EEC carcinogenesis and (2) genes relevant to ER signaling pathways [based on Kyoto Encyclopedia of Genes and Genomic (KEGG) estrogen signaling pathway (hsa04915)] (Kanehisa et al., 2016).

#### Sanger Sequencing for Validation

Identified variants were selected randomly for validation by Sanger sequencing. Primers for Sanger sequencing validation were designed using the IDT-DNA Primer Quest (Coralville, IA, United States). The PCR primers were described in **Supplementary Table S2**. PCR amplifications were performed using Applied Biosystem AmpliTaq^®^ Gold 360 master Mix (Applied Biosystems, Foster City, CA, United States) following manufacturer’s instructions. Sanger sequencing was performed using BigDye Terminator v.3.1 Cycle Sequencing kits (Applied Biosystems, Foster City, CA, United States) on an ABI 3500 Genetic Analyzer platform (Life Technologies, Guilford, CT, United States).

#### Site-Directed Mutagenesis and Constructs

The wild-type plasmid construct of *WHSC1* (RC212404) was obtained from OriGene Technologies (Rockville, MD, United States). Site-directed mutagenesis was performed using the Quick-Change^TM^ Site-Directed Mutagenesis Kit (Stratagene, La Jolla, CA, United States), according to the manufacturer’s instructions. The mutagenic primers were designed using QuickChange Primer Design Program and the sequences were provided in **Supplementary Table S3**.

#### Cell Lines and Transient Transfection

Endometrioid endometrial cancer-1 (ATCC^®^ CRL-2923^TM^) and 293T (ATCC^®^ CRL-3216^TM^) cells were maintained in RPMI 1640 and DMEM: F12 (both from Gibco, United States) supplemented with heat inactivated 10% fetal bovine serum (FBS; Gibco, United States). Twenty-four hours prior to transfection, cells were seeded in a 6-well plate with a density of 4 × 10^5^ (EEC-1) and 6 × 10^5^ (293T). Four micrograms of each construct was transfected using 8 μl Lipofectamine 2000 (Invitrogen, Carlsbad, CA, United States) according to the manufacturer’s instructions. Control cells were cells transfected with the empty vector pCMV6.

#### Gene Expression and Protein Analysis

Total RNA was isolated from the cell lines using the RNeasy Kit (Qiagen, Valencia, CA, United States) according to the manufacturer’s directions and cDNA was synthesized using High Capacity RNA to cDNA Kit (Applied Biosystem, Foster City, CA, United States). Quantitative real-time PCR was performed using TaqMan Fast Advanced Master Mix on the ABI 7500 Fast Real-Time PCR system (Applied Biosystem, Foster City, CA, United States). Relative expression was calculated using the ΔΔC_t_ method (Livak and Schmittgen, 2001) with GAPDH as the housekeeping gene.

Protein lysate was resolved on a mini-protean TGX precast gel of 4–20% polyacrylamide (BioRad Laboratories, Hercules, CA, United States) and analyzed against the following primary antibodies; anti-DDK (Clone OTI4C5) (OriGene Technologies, Rockville, MD, United States), NSD2 (G12), ERα (HC-20), and anti-β-actin (sc-47778) (Santa Cruz Biotechnology Inc., Santa Cruz, CA, United States).

#### Cell-Based Assays

Cell viability was assessed using PrestoBlue^®^ cell viability reagent (Invitrogen, Carlsbad, CA, United States), wound healing assay was performed using IBIDI Culture-Inserts (IBIDI GmbH, Martinsried, Germany), migration ability of the cells was assessed using QCM^TM^ 24-well cell migration assay (Millipore, Billerica, MA, United States), and colony formation assay was performed by seeding 500 transfected cells in six-well plates followed by incubation at 37°C for 14 days and crystal violet staining. All assays were performed in triplicate.

#### BrdU Proliferation Assay

Endometrioid endometrial cancer-1 cell lines were seeded at 2 × 10^4^ cells per well in 96-well plate and transfected with mutants or wild type of WHSC1 expression constructs before BrdU incorporation using BrdU Cell Proliferation Assay (Millipore, Temecula, CA, United States).

#### Luciferase Assay

To substantiate the importance of *WHSC1* as a potential coactivator, dual luciferase assay was performed. We used Cignal ERE Reporter Assay kit from SABiosciences (Valencia, CA, United States). 293T cells were plated at a density of 3 × 10^5^ cells per well (96-well plates) in phenol red-free DMEM: F12 with 10% v/v charcoal–dextran-treated FBS. The cells were then transfected with 100 ng of reporter plasmid, wild-type *WHSC1* plasmid, and Renilla. Each sample was done in triplicate. The *WHSC1* mutants were transfected in the same manner. After transfection for 24 h, the cells were stimulated with 10 nM E2 or vehicle (DMEM). The Dual-Glo Luciferase Assay System (Promega) was used according to the manufacturer’s instructions using VARIOLUX luminometer. Values were normalized to Renilla luciferase activity.

## Results

### Patient Demographic Data and Tumor Characteristic

Clinical specimens used in this study were collected from patients who were treated at the UKMMC. The information on the patient’s age, degree of differentiation, FIGO staging of tumor, metastasis, and ER status is summarized in **Table 1**. The majority of EEC in both groups were stage 1B 64% (7/11) in ER positive, 50% (4/8) in ER negative. While there are three cases of metastasis in ER negative = 37.5%, (3/8) has shown patients with advance disease.

**Table 1:** Patient demographics and tumor characteristics.

Characteristics	Classification	ER positive, *n* = 11	ER negative, *n* = 8
**Age**	**Average**	**52**	**57**
FIGO staging	IA	0 (0)	0 (0)
	IB	7 (64)	4 (50)
	IC	2 (18)	2 (25)
	IIA	1 (9)	0 (0)
	IIB	0 (0)	1 (12.5)
	IIIA	0 (0)	0 (0)
	IIIB	0 (0)	1 (12.5)
	N/A	1 (9)	0 (0)
Degree of differentiation	Grade 1	5 (45)	4 (50)
	Grade 2	4 (36)	2 (25)
	Grade 3	1 (9)	1 (12.5)
	N/A	1 (9)	1 (12.5)
Metastasis lymph nodes	Yes	0 (0)	3 (37.5)
	No	11 (100)	5 (62.5)

### Mutations Analysis of Endometrial Cancer With Ion Ampliseq^TM^ Cancer Panel

**FIGURE 1:**
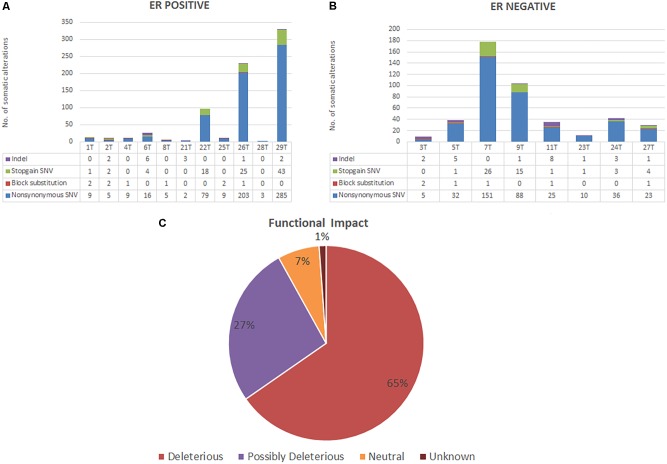
**(A,B)** Bar charts showing the number of somatic alterations identified in each patients according to respective groups. Indel in these bar charts including frameshift and non-frameshift substitution. **(C)** Pie chart showing the frequency of functional impact of gene mutations based on protein prediction algorithms in ANNOVAR.

All patients from both groups have at least three alterations among the 409 genes screened. A total of 741 variants were identified in 272 genes in ER positive subtype [718 single-nucleotide variations (SNVs), 14 insertions/deletions (INDELs), and 9 block substitutions] (**Figure 1A**). While in ER negative subtype, a total of 448 variants altered in 221 different genes, including 421 SNVs, 21 INDELs, and 6 block substitutions (**Figure 1B**). We noted that mutations distribution of EECs in both subtypes were genomically heterogeneous. There were five highly mutated patients (median 178 per tumor, range from 97 to 330) and another 14 patients were non-highly mutated (median 11 mutations per tumor; range 3–42). We further investigated and found that hypermutated phenotype group could have a perturbed DNA repair system due to frequently altered DNA repair genes especially in MMR genes such as (i.e., *MSH2*, *MSH6*, and *PMS1*) (**Supplementary Table S4**).

Based on protein prediction algorithms among identified 1139 SNVs in both subtypes, 739 variants (65%) were predicted to have deleterious effects, 301 variants (27%) have possibly deleterious effects, while the other 78 variants (7%) has neutral or low protein impact (**Figure 1C**). We identified 134 out of 371 (36%) genes predicted as tumor driver which included a total of 295 alterations. From this 295 alteration, 178 (60%) were identified in ER positive whereby 9 out of 11 ER positive patients have at least two candidate driver alteration. Meanwhile in ER negative subtype, 117 (40%) alterations were identified in ER negative patients where all ER negative patients have at least three candidate driver alterations (**Supplementary Table S5**).

**FIGURE 2:**
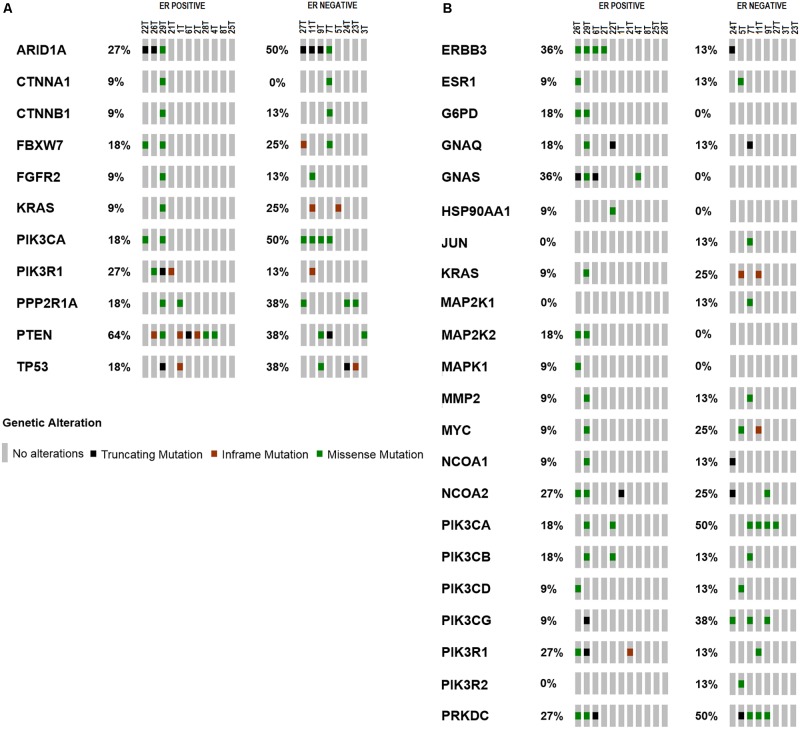
Frequency of mutations in **(A)** genes related in carcinogenesis of EEC. *PTEN* is the most frequently altered gene in ER positive subtype (64%, *n* = 11); *ARID1A* is the most frequently altered gene in ER negative group (63%, *n* = 8). **(B)** Frequency of mutations in genes relevant to ER signaling pathway compiled from literatures. *ERBB3* and *GNAS* are the most frequently altered genes in ER positive subtype (36%, *n* = 11); PIK3CA is the most frequently altered gene in ER negative group (50%, *n* = 8).

PTEN is the most frequently altered gene in ER positive subtype (64%, 7/11) while ARID1A is the most frequently altered gene in ER negative group (50%, 4/8) as shown in **Figure 2A** (**Supplementary Tables S6**, **S7**). Majority of *PTEN* alterations were missense and in-frame mutations. On the other hand, *ARID1A* mutations were truncating alterations including nonsense mutations and INDELs. Mutations in the ER signaling pathway is known to be important in endometrial cancer; therefore, we further examined the prevalence of mutations in the genes involved in ER signaling pathway. Overall, there are 38 genes out of 409 genes panel list of CCP known to be involved in ER signaling pathway (**Supplementary Table S1**). As shown in **Figure 2B** (**Supplementary Tables S8**, **S9**), ERBB3, GNAS, PIK3R1, and WHSC1 are the most frequently altered genes in ER positive subtype (36%, 4/11); (36%, 4/11); (27%, 3/11); and (27%, 3/11) while PIK3CA is the most frequently altered gene in ER negative group (50%, 4/8). **Table 2** showed classification based on protein impact prediction and candidate driver mutations for each alteration in *PTEN*, *ARID1A*, *PIK3CA*, *ERBB3*, *GNAS*, *PIK3R1*, and *WHSC1*.

**Table 2:** Classification of somatic alterations in PTEN, PIK3CA, ARID1A, ERBB3, GNAS, PIK3R1, and WHSC1 genes.

Patient	ER subtype	Gene	Exonic function	Protein change	Exon	Protein impact prediction	Driver prediction
IT	ER + VE	PTEN	Nonframeshift substitution	R130P	5	NA	Driver
2T	ER + VE	PTEN	Nonframeshift substitution	R130G	5	NA	Driver
4T	ER + VE	PTEN	Nonsynonymous SNV	K128T	5	Deleterious	Driver
6T	ER + VE	PTEN	Frameshift deletion	R11fs	1	NA	Driver
26T	ER + VE	PTEN	Nonframeshift substitution	R130Q	5	NA	Driver
28T	ER + VE	PTEN	Nonsynonymous SNV	P190L	6	Deleterious	Passenger
29T	ER + VE	PTEN	Nonsynonymous SNV	F341V	8	Deleterious	Driver
29T	ER + VE	PTEN	Nonsynonymous SNV	D92E	5	Deleterious	Driver
7T	ER - VE	PTEN	Stopgain SNV	S59X	3	Deleterious	Driver
7T	ER - VE	PTEN	Stopgain SNV	E7X	1	NA	Driver
9T	ER - VE	PTEN	Nonsynonymous SNV	F145V	5	Deleterious	Driver
24T	ER - VE	PTEN	Nonsynonymous SNV	I122N	5	Deleterious	Driver
22T	ER + VE	PIK3CA	Nonsynonymous SNV	T1025A	21	Possibly deleterious	Driver
22T	ER + VE	PIK3CA	Nonsynonymous SNV	P449T	8	Deleterious	Driver
29T	ER + VE	PIK3CA	Nonsynonymous SNV	E453K	8	Possibly deleterious	Driver
29T	ER + VE	PIK3CA	Nonsynonymous SNV	E365K	6	Possibly deleterious	Driver
7T	ER - VE	PIK3CA	Nonsynonymous SNV	E81K	2	Deleterious	Driver
7T	ER - VE	PIK3CA	Nonsynonymous SNV	Q879K	18	Possibly deleterious	Passenger
7T	ER - VE	PIK3CA	Nonsynonymous SNV	I816T	17	Deleterious	Passenger
7T	ER - VE	PIK3CA	Nonsynonymous SNV	R54K	2	Possibly deleterious	Passenger
9T	ER - VE	PIK3CA	Nonsynonymous SNV	G118D	3	Deleterious	Driver
11T	ER - VE	PIK3CA	Nonsynonymous SNV	Q60H	2	Possibly deleterious	Passenger
27T	ER - VE	PIK3CA	Nonsynonymous SNV	M1043V	21	Deleterious	Driver
22T	ER + VE	ARID1A	Stopgain SNV	S2269X	20	Deleterious	Passenger
26T	ER + VE	ARID1A	Stopgain SNV	R1989X	20	Deleterious	Driver
26T	ER + VE	ARID1A	Nonsynonymous SNV	R1636W	18	Deleterious	Driver
29T	ER + VE	ARID1A	Nonsynonymous SNV	P1576L	18	Deleterious	Passenger
7T,9T	ER - VE	ARID1A	Stopgain SNV	R1989X	20	Deleterious	Driver
7T	ER - VE	ARID1A	Nonsynonymous SNV	S1985P	20	Deleterious	Passenger
9T	ER - VE	ARID1A	Frameshift deletion	G2040fs	20	NA	Driver
11T	ER - VE	ARID1A	Frameshift deletion	G324fs	1	NA	Driver
27T	ER - VE	ARID1A	Stopgain SNV	R1335X	16	Deleterious	Driver
2T	ER + VE	ERBB3	Nonsynonymous SNV	P558S	14	Deleterious	Passenger
2T	ER + VE	ERBB3	Nonsynonymous SNV	R488Q	12	Deleterious	Passenger
6T	ER + VE	ERBB3	Nonsynonymous SNV	L924V	23	Deleterious	Driver
26T	ER + VE	ERBB3	Nonsynonymous SNV	L143M	4	Deleterious	Driver
29T	ER + VE	ERBB3	Nonsynonymous SNV	G1073V	27	Possibly deleterious	Passenger
29T	ER + VE	ERBB3	Nonsynonymous SNV	R670Q	17	Neutral/Benign	Passenger
4T	ER - VE	ERBB3	Frameshift deletion	E560fs	14	NA	Passenger
6T	ER + VE	GNAS	Stopgain SNV	S192X	1	Possibly deleterious	NA
26T	ER + VE	GNAS	Stopgain SNV	R327X	11	Possibly deleterious	Passenger
29T	ER + VE	GNAS	Nonsynonymous SNV	I39T	1	Deleterious	NA
29T	ER + VE	GNAS	Nonsynonymous SNV	P338L	1	Neutral/benign	NA
4T	ER + VE	GNAS	Nonsynonymous SNV	R194P	1	Neutral/benign	NA
21T	ER + VE	PIK3R1	Nonframeshift deletion	578_580del	13	NA	Passenger
26T	ER + VE	PIK3R1	Nonsynonymous SNV	M525I	13	Possibly deleterious	Passenger
29T	ER + VE	PIK3R1	Stopgain SNV	R348X	9	Deleterious	Driver
29T	ER + VE	PIK3R1	Stopgain SNV	R162X	4	Deleterious	Driver
11T	ER - VE	PIK3R1	Nonframeshift deletion	404_405del	10	NA	Passenger
22T	ER + VE	WHSC1	Nonsynonymous SNV	K547E	7	Deleterious	Driver
22T	ER + VE	WHSC1	Stopgain SNV	R824X	13	Deleterious	Passenger
26T	ER + VE	WHSC1	Nonsynonymous SNV	R1126H	19	Deleterious	Driver
29T	ER + VE	WHSC1	Nonsynonymous SNV	L1268P	21	Deleterious	Driver
9T	ER - VE	WHSC1	Nonsynonymous SNV	P748L	12	Deleterious	Driver

### Mutations Distribution in the Functional Domains of *PTEN*, *ARID1A*, *ERBB3*, and *WHSC1*

Mutations distribution in the functional domains of *PTEN*, *ARID1A*, and *WHSC1* was illustrated in **Supplementary Figure S2**. Mutations in *PTEN* were localized to the catalytic phosphatase domain, with majority of mutants belongs to ER positive subtype group including the recurrent hotspot R130 residue, D92E, K128T (**Supplementary Figure S2a**). In addition, a frameshift deletion (R11fs) and two missense mutations (P190L and F341V) in C2 domain were also found in ER positive subtype. While in ER negative subtype group, two nonsense mutations (E7X and S59X) and two missense alterations (I122N and F145V) located on phosphatase domain have been identified. Most of these mutations have been reported via COSMIC v82, with the exception to R11fs, which was identified in our study.

Out of the 19 EECs sequenced, seven patients had nine *ARID1A* mutations (four mutations were identified in ER positive and five in ER negative EEC patients). Interestingly, recurrent hotspot mutation (R1989X) was observed in both ER positive and ER negative subtype group as shown in **Supplementary Figure S2b**. More *ARID1A* mutations were identified among ER negative subtype group, with mainly truncating type of mutations (nonsense and frameshift deletion) (G324fs, R1335X, R1989X, and G2040fs) and a missense mutation (S1985P). However, two nonsense mutations (R1989X and S2269X) and two missense mutations (P1576L and R1636W) which were located on non-functional domain of *ARID1A* have been identified in ER positive subtype. This study is the first to report the somatic G2040fs variant in EEC.

Higher mutation frequency of *ERBB3* was observed in ER positive subtype (36%, 4/11) compared to ER negative subtype (18%, 1/8). As shown in **Supplementary Figure S2c**, the missense mutations in ER positive sample were mapped at functional domain of ERBB3 gene including two in extracellular domain (L143M and P558S) and one in tyrosine kinase domain (L924V), while the others mapped on non-functional domain (R488Q), (R670Q), and (G1073V). Nevertheless, the single frameshift deletion mutation (E560fs) reported in ER negative subtype was located at the extracellular domain IV of *ERBB3*. To date, most of the mutations we discovered in ERBB3 gene are novel, except for L143M, R488Q, and R670Q that have been recorded in Cosmic v82 and ICGC database, respectively.

*WHSC1* was found to be altered in three ER positive patients and one ER negative patient. Majority of these somatic variations were distributed along the functional domain of *WHSC1* as shown in **Supplementary Figure S2d**. All of the variants identified in this study are novel, with the exception of a mutation on the catalytic SET domain (R1126H) that has been identified in several cancers (**Supplementary Table S10**). One missense mutation K547E was identified in an area without a defined domain, while two missense mutations (P748L and L1268P) were located within the plant homeodomain (PHD) domains II and V, respectively. A nonsense mutation (R824X) was identified which was predicted to cause premature truncation of WHSC1 protein with a deletion of C terminal functional domains including PHD IV, proline–tryptophan–tryptophan–proline-2 (PWWP2), SET, and PHD V-CH5CH domain. The frequency and location of alterations in *WHSC1* suggest that these cancer-associated mutations may have functional consequences.

### Expression Analysis of Wild-Type and Mutant Constructs

**FIGURE 3:**
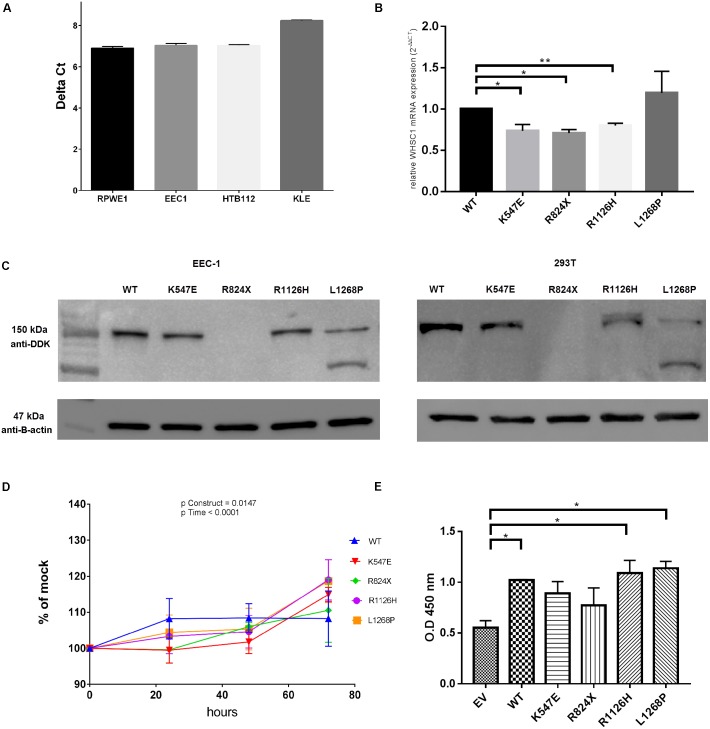
WHSC1 gene and protein analysis. **(A)** Quantification of mRNA from nuclear extract of normal prostate cell lines RPWE1 and endometrial cancer cell lines (EEC-1, HTB-112, KLE). **(B)** Quantification of mRNA expression of mutant *WHSC1* in EEC-1 cell line. Relative WHSC1 mutants were calculated in comparison to WT *WHSC1* 48 h post-transfection by real-time PCR and normalized to GAPDH mRNA expression. Negative control (no template) was included in each experiment (mean ± SD, *n* = 2, ^∗^*p* < 0.05). The mRNA expression of WT was assigned as 1. **(C)** Western blot analysis shows protein expression of wild type and *WHSC1* mutants in EEC-1 and 293T cell lines after 48 h post-transfection. Total protein harvested from EEC-1 cells and 293T cells solubilized in cell lysis buffer were analyzed using precast SDS 4–12% gels. The WT and mutants of WHSC1 (152.1 kDa) were detected using Anti-DDK Clone OTI4C5 (mouse origin). While β-actin (47 kDa) was detected using anti-β-actin (mouse origin) for positive control. Mutants *WHSC1* promote cell growth. **(D)** Mutants *WHSC1* promote cell viability. At 72 h, the mutants K547E, R1126H, and L1268P resulted in 15, 19, and 18% increase significantly in motility, respectively, over EV in EEC-1. Statistical significance in all cases was measured by Student’s *t*-test (^∗^*p* < 0.05), *n* = 3. Error bars represent average ± SD. Overall *p*-values were calculated by two-way ANOVA for time. **(E)** Mutants *WHSC1* promote cell proliferation. BrdU colorimetric incorporation assay shows that *wild type* of *WHSC1*, R1126H, and L1268P significantly increased cell proliferation in EEC-1. Statistical significance in all cases was measured by Student’s *t*-test (^∗^*p* < 0.05), *n* = 2. Error bars represent average ± SD.

The level of *WHSC1* expression was firstly screened in several cell lines (**Figure 3A**) in order to gauge its expression. Upon transfection, *WHSC1* mRNA levels were significantly reduced in the transfected cells with the mutant constructs K547E, R824X, and R1126H compared to the wild type indicating that these mutations affected the stability of the mRNA levels. However, the wild type and mutant L1268P were equally expressed in the transfected EEC-1 cell line (**Figure 3B**). This result was confirmed by the Western blot in EEC-1 and 293T cell lines (**Figure 3C**). Of note however, there is a second migrating band seen in L1268P in Western blot assay, which might explain the differences of mRNA of transcription level of L1268P and other mutant constructs.

### Assessment of Oncogenic Properties of *WHSC1* Mutants

At 72 h, the mutants K547E, R1126H, and L1268P showed a 15 (*p* = 0.0002), 19 (*p* = 0.0043), and 18% (*p* = 0.0056) significant increase in cell proliferation, respectively, over the empty vector control in EEC-1 cell line (**Figure 3D**). The effect of the *WHSC1* mutants on cell proliferation was further assessed by quantifying the activity of proliferating cells within 24 h after BrdU labeling. BrdU incorporation assay revealed significant increase in DNA synthesis in *WHSC1* wild type (*p* = 0.0190), R1126H (*p* = 0.0341), and L1268P (*p* = 0.0136) mutants, relative to empty vector. In contrast, no significant effect on DNA synthesis was caused by the K547E and R824X mutants (**Figure 3E**).

**FIGURE 4:**
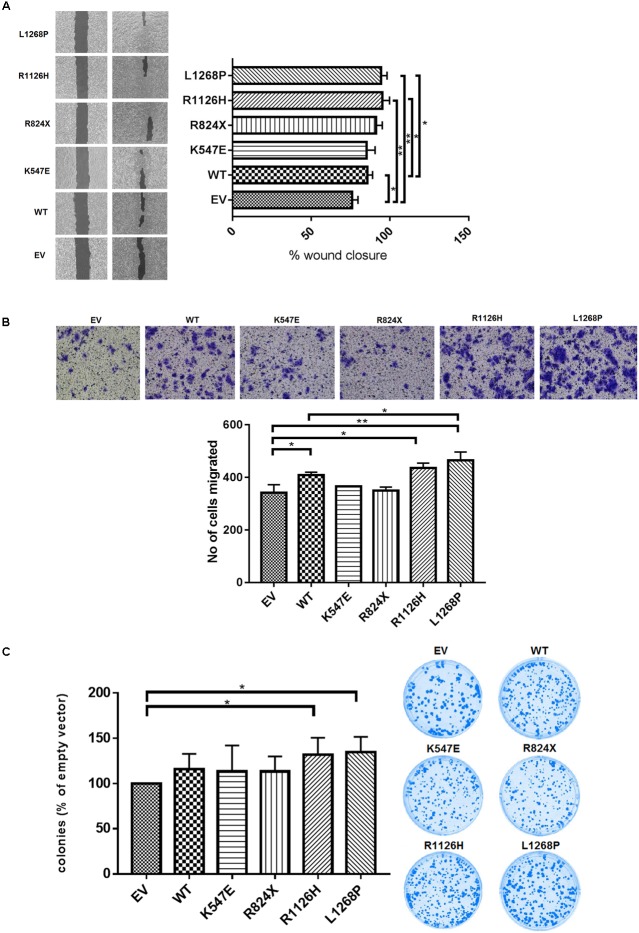
Effect of *WHSC1* mutants on cell migration and clonogenic potential. **(A)** Wound healing assay displayed significant increase in average rate of wound closure at 72 h shown in R1126H and L1268P compared with EV and WT. **(B)** Transwell migration assay showed the numbers of migrated mutants R1126H and L1268P were significantly higher than EV. **(C)** Clonogenic survival was assessed by colony formation assay at day 14. The numbers of colonies were counted after crystal violet staining, and are expressed as percentage of cells expressing empty vector. There is significance of colony formation in R1126H and L1268P when compared with empty vector. No obvious effects were observed on the colony formation ability of EEC-1 cells after transfection with WT and mutants K547E and R824X. Statistical significance was measured by Student’s *t*-test (^∗^*p* < 0.05), *n* = 3. Error bars represent average ± SD.

To further address the role of constitutive activation of *WHSC1* mutants on cell migration, we employed both the wound-healing and transwell migration assays. As shown in **Figure 4A**, the open area was rapidly covered by the mutant R1126H and L1268P in comparison with control (*p* = 0.0049 and *p* = 0.0040, respectively). The quantified open area in vector control cells reduced from 100 to only 76%, while R1126H mutant cells reduced from 100 to 95%, whereas L1268P mutant caused reduction from 100 to 94%. The wild type *WHSC1* also showed significant wound closure from 100 to 85% (*p* = 0.0233). To further confirm migration ability of the mutants, we employed the transwell migration assay. Similarly, EEC-1 transfected with *WHSC1* mutants R1126H and L1268P displayed a significant increase in number of migrated cells (**Figure 4B**) (*p* = 0.0102 and *p* = 0.0086, respectively). In this transwell migration assay, the wild-type *WHSC1* also showed potential migration ability compared with vector alone control cells (*p* = 0.0233). In addition, only mutants R1126H and L1268P showed a significant increase in colony number compared to empty vector (**Figure 4C**) (*p* = 0.00416 and *p* = 0.0232, respectively).

### *WHSC1* Regulates ERα Gene Expression

**FIGURE 5:**
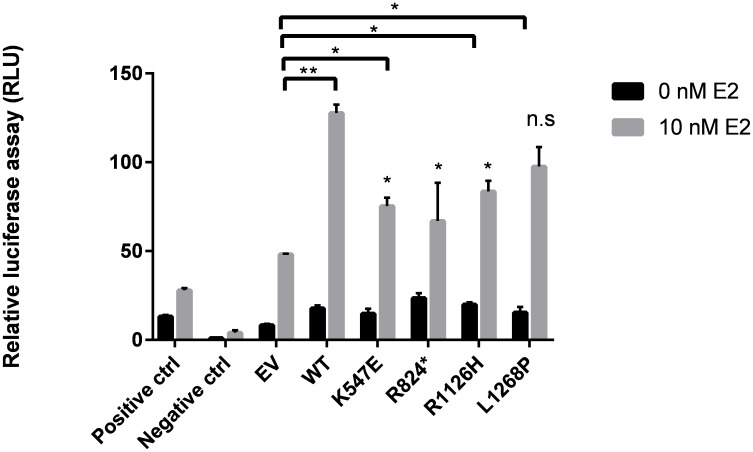
Effects of *WHSC1* mutations on transcriptional activation of Estrogen Receptor Gene (ERα). WT *WHSC1* showed increase in ERE-mediated luciferase activity relative to EV. K547E, R824X, and R1126H were associated with reduced ability to activate ER activity, compared to WT. Whereas L1268P activated ER activity comparable to WT (n.s.). Data are presented as mean values ± SD, *n* = 2.

Both R1126H and L1268P led to a significant increase of the coactivation potential in *WHSC1* (**Figure 5**) albeit lower than the wild type *WHSC1* when compared with empty vector. However, the truncated R824X mutant showed reduction of the coactivation potential of *WHSC1* suggesting that the catalytic SET activity is required for its ability to stimulate estrogen-dependent gene transcription. When compared all mutants with the wild type, only L1268P signaling did not differ significantly.

### Activating *WHSC1* L1268P Is Sensitive to Fulvestrant

**FIGURE 6:**
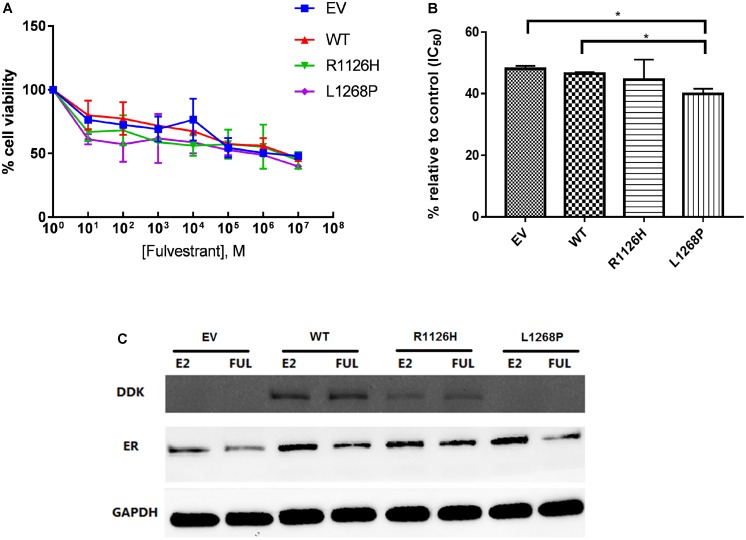
Effect of Fulvestrant on cell proliferation in mutants *WHSC1* expressing EEC1 cells. **(A)** Treatment was performed with 10 nM E2 and various dosage of Fulvestrant for 24 h. **(B)** Quantification of proliferation after exposure to 10 nM E2 and 10^-3^ M Fulvestrant. The values represent the percentage of growing cells compared to the normalized control (100%). Results represent mean values ± SD, *n* = 2 performed in triplicate. *WHSC1* mutants counteract the effect of ER inhibitor. **(C)** EEC-1 cells were transiently transfected with WT, R1126H, and L1268P mutant constructs and treated with 10 nM E2 or 100 nM of Fulvestrant for 24 h. Protein lysates were prepared and analyzed by Western Blot with indicated antibodies.

Fulvestrant acts as an antagonist to inhibit ER activity; therefore, we examine the effect of *WHSC1* R1126H and L1268P on the dose-dependent inhibition of proliferation in EEC-1 cells. Fulvestrant was able to inhibit the activity of wild type and *WHSC1* mutants. As shown in **Figures 6A,B**, L1268H had significantly greater antiproliferative effect only when compared with the empty vector with a 60% growth inhibition (*p* = 0.0281) compared to the wild type (54%) which was not significant (*p* = 0.362). The estrogen responsiveness was assayed as shown in **Figure 6C**, where we compared the ability of Fulvestrant to reduce ER protein levels in wild-type and mutants *WHSC1*, in the presence and absence of estrogen. We observed that Fulvestrant was able to downregulate the ER proteins of both wild type and mutants, with more reduction seen in the L1268P mutant’s ER proteins level. Upon treatment with estradiol and Fulvestrant, a clear reduction of steady-state L1268P *WHSC1* mutant’s exogenous protein can be seen in the Western blot assay.

## Discussion

Over the past few years emergence of novel technologies based on NGS have led to a new paradigm in understanding the mutational landscape of endometrial cancers. However, most studies were limited to either characterization of endometrial cancer without stratification based on grade and histological subtypes, or were focused on aggressive serous carcinoma subtype of endometrial cancer only (Kuhn et al., 2012; Le Gallo et al., 2012; Liang et al., 2012; Chang et al., 2017). Our study is not only the first report on the mutational landscape in ER positive and ER negative EEC, but also on the functional analysis of a potential gene candidate, *WHSC1*, involved in ER positive endometrioid tumorigenesis. Of the 11 ER positive and 8 ER negative EEC patients sequenced in our study, the mutation spectrum notably diverges among both subtypes.

Among known genes that have been reported to be altered in endometrial cancer in earlier studies, *PTEN* mutations occur frequently in ER positive subtype compared to ER negative subtype. In the TCGA study on EEC, *PTEN* mutations have also been associated with ER expression in endometrial cancer (The Cancer Genome Atlas Research Network et al., 2013). However, our study showed that *PIK3CA* mutations were comparable between both ER subtypes with more events occurring in the ER negative subtype compared to ER positive subtype. This is inconsistent with results from TCGA on breast cancer, which suggested the association between *PIK3CA* with ER positive expression (The Cancer Genome Atlas Network, 2012). From the 10 patients with *PTEN* mutation in our study, only 3 patients had mutation in *PIK3CA*, which is consistent with other investigators that claimed *PIK3CA* mutations are mutually exclusive with *PTEN* mutations, suggesting that carcinogenic signaling through phosphatidylinositol 3-kinase (PI3K) pathway is mediated either through inactivation of *PTEN* or activation of *PIK3CA* (Prat et al., 2007). *ARID1A* mutations were identified in both endometrial cancer subtypes but more commonly seen in the ER negative subtype. Almost all *ARID1A* mutants in our analysis coexist with *PTEN* and *PIK3CA* mutations, and it is postulated that these mutations together induce aberrant activation of PI3K and protein kinase B (AKT) phosphorylation that result in inhibition of cell survival and apoptosis (Liang et al., 2012).

PTEN is a 200-kb gene located on chromosome 10q23.3 that consists of nine exons coding for 403 amino acids yielding a 47 kDa protein (Li, 1997). This tumor suppressor gene plays an important role in the tumorigenesis of endometrial cancer, particularly in EEC (Djordjevic et al., 2012; O’Hara and Bell, 2012; The Cancer Genome Atlas Research Network et al., 2013). *PTEN* functions primarily as a lipid phosphatase via a dual specificity protein phosphatase dualistic domain (Maehama and Dixon, 1998). Remarkably, most reported mutations (R130, I122N, K128T) are clustered within the phosphatase signature motif between codon 122–132 that has been considered as the mutation hotspot site (Prasad et al., 2005; Bansal et al., 2009). In addition, mutations on the phosphatase binding loop D92E on this domain play critical roles as well (Giudice and Squarize, 2013). These mutants may abolish the phosphatase activity of the substrate phosphoinositide lipid, a key signaling component of PI3K pathway that regulate cell growth, proliferation, migration, and apoptosis (Fayard et al., 2010). In comparison, mutants located on C2 domain (P190L and F341V) could impair the phosphoinositide lipid binding function, ultimately its growth-suppressing activity despite retaining the catalytic phosphatase activity (Naguib et al., 2015). Other truncating mutations including nonsense and frameshift deletion (E7X, S59X, and R11fs) may result in functional inactivation of PTEN protein function, which leads to tumorigenesis (Xu et al., 2014). A number of *PTEN* missense mutations have been screened for its phosphatase activity which is important for its tumor suppression function, and majority of the mutants were shown to eliminate or reduce phosphatase activity (Han et al., 2000).

*ARID1A* mutations have been increasingly reported upon the emergence of NGS technology especially among various malignancies (Jones et al., 2012) including endometrial cancer (Liang et al., 2012). *ARID1A* is located on chromosome 1p36.11 and it contains 20 coding exons. The gene encodes a 24-kDa chromatin remodeling protein consisting of 2285 amino acids and a member of SWI/SNF family which plays a significant role in regulating the transcription of genes via ATP hydrolysis (Wilson and Roberts, 2011). Therefore, it is critical in regulating diverse cellular processes including DNA repair, differentiation, and development as well as tumor suppression (Lu and Allis, 2017). The *ARIDIA* mutations identified in our study are consistent with others regardless of the type of cancers, where most were truncating mutations that would result in loss of function, supporting its tumor suppressor role (Guan et al., 2012; Chang et al., 2015). Both nonsense and frameshift mutants (R1335X, R1989X, S2269X, G324fs, and G2040fs) would result in loss of function *ARID1A* protein by nonsense-mediated mRNA decay and degradation of misfolded truncated proteins. Thus, its tumor suppression activity is abolished due to disruption in CDKN1A and SMAD3 pathway which is related to cell cycle regulation and aberrant activation of PI3K/AKT pathway (Bosse et al., 2013). Missense mutations occur less frequently in *ARID1A*; hence, the functional manifestations are poorly understood (Bateman et al., 2016). Our study showed that the mutations commonly occurred in the ER negative subtype group, suggesting that inactivation of *ARID1A* might play important role in ER-mediated transcription in endometrial cancer. Moreover, the SWI/SNW complex has been reported to be vital for the transcriptional activation of ER (Belandia et al., 2002). Mutation in *ARID1A* is not that frequent in breast cancer (only ∼5%) (Jones et al., 2012) and there has been no reported association with ER status. However, low *ARID1A* expression has been found to associate with ER negativity of breast cancer (Zhang et al., 2012).

By focusing on genes involved in the ER signaling pathway, we identified relatively frequent mutations in *ERBB3*, *GNAS*, *PI3KR1*, and *WHSC1* in ER positive subtype. Previous data by TCGA reported that 5.2% (16/373) of EEC have *ERBB3* mutations (Cerami et al., 2012). This gene is located on 12q13.2 chromosome, consists of 28 exons, and encodes a protein composed of 1342 amino acids (Sithanandam and Anderson, 2008). *ERBB3* encodes a member of EGFR family receptor tyrosine kinase, which is a well-known gene to be amplified and overexpressed in various cancers (Olayioye et al., 2000). With emergence of NGS, more somatic mutations in *ERBB3* have gained attention. ERBB3 has been uncovered as a driver cancer gene in endometrial cancer through whole exome sequencing and loss of function screening by Liang et al. (2012). Using whole exome sequencing, *ERBB3* mutations have been reported in colon, gastric, and gallbladder cancers (Jaiswal et al., 2013; Li et al., 2014). *ERBB3* comprises of extracellular ligand-binding domains (I–IV), a tyrosine kinase domain, and a regulatory domain (Olayioye et al., 2000). In concordance with previous studies, the majority of mutations detected in our patients are located in the extracellular domain (L143M, P558S, E560fs) of the gene which are predicted to induce conformational changes, dysregulate ligand binding, or heterodimerization of *ERBB3* with kinase-active *ERBB2* (Jaiswal et al., 2013). Distinct from other EGFR members, *ERBB3* has impaired tyrosine kinase activity; hence, it could be hypothesized that mutations mapped on this domain (L924V) could activate and stimulate protein tyrosine kinase (Jeong et al., 2006). These oncogenic mutations might promote tumorigenesis through aberrant activation of PI3K/AKT pathway, eliciting various biological responses including cell cycle progression, stimulation of cell migration, and invasion (Sithanandam and Anderson, 2008).

Perhaps the most promising gene from our study, based on mutation frequency and function, is WHSC1. It was more frequently mutated in the ER positive subtype. The WHSC1 gene encodes a histone methyltransferase, is classified as chromatin modification group, and plays an important role in regulating gene transcription (Li et al., 2009). A previous study showed that *WHSC1* interacts with and regulate gene transcription activity of androgen receptors in prostate cancer (Kang et al., 2009) and ER in breast cancer (Feng et al., 2014), which provide a new basis for tumorigenesis in endocrine-related cancers. Hence, there is high possibility that *WHSC1* is involved in development of endometrial cancer. *WHSC1* is shown to be essential for ERα-dependent transcription induction in tamoxifen-resistant breast cancers, one of the potent coactivators in the ER pathway (Feng et al., 2014). They postulated that *WHSC1* is recruited to the ER promoter by the bromodomain protein (BRD4) and direct H3K36 methylation that is responsible for promoting transcription initiation and elongation. Moreover, genetic alterations in the transcriptional coregulators of ER genes are likely to be the key players in the development of estrogen-dependent tumors through deregulation in the estrogen signaling pathway (Girault et al., 2006), further supporting our hypothesis for *WHSC1* as the likely target gene in endometrial cancer.

*WHSC1* (MMSET/NSD2) spans 120 kb, consists of 24 exons that encode a 1360 amino acid protein containing catalytic SET domain, and several chromatin-associated domains comprising a high mobility group (HMG) box which often representing a DNA-binding domain, two PWWP domains, five PHD zinc fingers defined as binding modules for methylated lysines, and C5HCH domain (Cys–His-Rich). Previous studies have almost exclusively focused on translocation and overexpression of *WHSC1* while mutations in this gene have not yet been extensively characterized (Jaffe et al., 2013), even though the occurrence in a variety of cancers is increasingly reported (Huether et al., 2014; García-Carpizo et al., 2016). Based on our analysis, *WHSC1* is frequently mutated in the ER positive subtype where four mutations (K574E, R1126H, R824X, and L1268P) were identified in three patients. We went on to study the role of these mutations in cell transformation by performing transient transfection in EEC-1 cell lines (endometrial cancer cell line expressing ER), followed by luciferase-based assay. Of the four mutations, we observed that only two *WHSC1* mutations (R1126H and L1268P) resulted in increased cell proliferation, increased ability of the cells to migrate, and survive in clonogenic growth which represents gain of function phenotype. Both mutants are likely to exhibit increased level of methyltransferase activity of H3K36, which is generally thought to drive tumorigenesis, through activation of key genes (Rao et al., 2005). We postulate that the mutant R1126H which is located in the histone binding groove on SET domain, which is in proximity to the established hotspot mutants E1099K and D1125N, lead to a hyperactive *WHSC1*, causing an increase in methyltransferases activity and hence progression of malignancies (Jaffe et al., 2013). Since the mutant R1126H exhibited independent ER pathway activation, it may be involved in endometrial carcinogenesis through other pathways. Endometrial cancer may also be triggered through the transcriptional regulation of CCND1, the target gene of the β-catenin/Tcf-4 complex, through H3K36 methylation and activation of the WNT signaling pathway (Toyokawa et al., 2011). This was supported by the discovery of recurrent R1126H mutants in colorectal cancer (**Supplementary Table S10**), where WNT signaling is the hallmark among the many genetic events. The other pathway that this mutation might be involved in is the RAS pathway by altering the oncogenic RAS transcriptional responses resulting in increased methyltransferase activity (García-Carpizo et al., 2016). However, there is still the need to elucidate which one is the key event since epigenetic alterations usually mediate the transcription of a plethora of genes (Huang et al., 2013).

Hormonal therapy and chemotherapy are frequently used for advanced endometrial cancer, and there is no approved molecular targeted therapy so far (Temkin and Fleming, 2009). However, in one clinical trial, the use of Fulvestrant has resulted in positive response in some patients with advanced or recurrent endometrial cancer (Emons et al., 2013). Fulvestrant is a selective ER downregulator (SERD) which competitively inhibits the binding of estradiol to the ER and it degrades ER via the ubiquitin-proteasome (Berry et al., 2008). Our study also provides evidence that Fulvestrant has the ability to induce ER degradation and that it inhibits the proliferation of the transforming mutant L1268P cells, which is associated with increased sensitivity to this treatment. Hence, we propose that Fulvestrant the potential to inhibit the activity of ER signaling pathway in this particular mutant.

## Conclusion

Using NGS, we have characterized the mutational landscape of different ER subtypes of EEC. We further demonstrated the role of the *WHSC1* L1268P mutation in endometrial cancer carcinogenesis by activating the ER signaling pathway and its pivotal role in promoting cell proliferation, migration, and survival. The ER signaling pathway activation by *WHSC1* LP1268H provides new evidence which is important for the future applications of targeted therapies for endometrial cancer carrying the WHSC1 L1268P mutations. A major limitation of our study is the small sample size, therefore further studies are warranted to explore the diagnostic and therapeutic potential of our discovery.

## Ethics Statement

This study was carried out in accordance with the recommendations of Guidelines for ethical review of clinical research or research involving human subjects. The protocol was approved by the Universiti Kebangsaan Malaysia Research Ethics Committee (UKM 1.5.3.5/244/AP-2012-011). All subjects gave written informed consent in accordance with the Declaration of Helsinki.

## Author Contributions

SSS performed the experiments and data analysis. N-SAM was involved in data interpretation, drafting the manuscript, and overseeing the experiments. SKS was heavily involved in data analysis. SES was involved in optimization of library preparation and sequencing. NA gave insight for the functional analyses. AMD is an endometrial surgeon involved in specimen collection and RMZ is a pathologist. RJ was involved in critical review of the manuscript. All authors read and approved the final manuscript.

## Conflict of Interest Statement

The authors declare that the research was conducted in the absence of any commercial or financial relationships that could be construed as a potential conflict of interest.

## Supplementary Material

FIGURE S1 Variant analysis and prioritization workflow. Summary of our variant evaluation process for identifying candidate mutations.

FIGURE S2 Mutations distribution across exon and functional domain of **(a)** *PTEN*, **(b)** *ARID1A*, **(c)** *ERBB3*, and **(d)** *WHSC1*. Any position with a mutation obtains a circle; the length of the line depends on the number of mutations detected at that codon. The gray bar represents the entire protein with the different amino acid positions (aa). The colored boxes are specific functional domains. On top of the lollipops variants are annotated as the amino-acid change at that specific site. Blue letter indicates mutation occur in ER positive subtype while green letter indicate mutation occur in ER negative subtype of our analysis.

TABLE S1 Ion Ampliseq^TM^ comprehensive cancer panel.

TABLE S2 Primer’s sequence used for Sanger validation.

TABLE S3 WHSC1 mutant primer for site-directed mutagenesis.

TABLE S4 Somatic alterations among DNA repair gene in hypermutated patient sample.

TABLE S5 Somatic alteration identified as driver mutations by Cancer Genome Interpreter.

TABLE S6 Somatic alterations of ER positive subtypes in endometrioid endometrial cancer-related genes.

TABLE S7 Somatic alteration of ER negative subtypes in endometrioid endometrial cancer-related genes.

TABLE S8 Somatic alterations of ER positive subtype in genes relevant to estrogen signaling pathway.

TABLE S9 Somatic alterations of ER negative subtype in genes relevant to estrogen signaling pathway.

TABLE S10 Recurrent mutation site of WHSC1 in different cancers.

## References

[B1] Amant F. Moerman P. Neven P. Timmerman D. Van Limbergen E. Vergote I. (2005). Endometrial cancer. *Lancet* 366 491–505. 10.1016/S0140-6736(05)67063-8 16084259

[B2] Backes F. J. Walker C. J. Goodfellow P. J. Hade E. M. Agarwal G. Mutch D. (2016). Estrogen receptor-alpha as a predictive biomarker in endometrioid endometrial cancer. *Gynecol. Oncol.* 141 312–317. 10.1016/j.ygyno.2016.03.006 26957478 PMC4878441

[B3] Bansal N. Yendluri V. Wenham R. M. (2009). The molecular biology of endometrial cancers and the implications for pathogenesis, classification, and targeted therapies. *Cancer Control* 16 8–13. 10.1177/107327480901600102 19078924

[B4] Bateman N. W. Shoji Y. Conrads K. A. Stroop K. D. Hamilton C. A. Darcy K. M. (2016). Identification and functional characterization of a novel bipartite nuclear localization sequence in ARID1A. *Biochem. Biophys. Res. Commun.* 469 114–119. 10.1016/j.bbrc.2015.11.080 26614907

[B5] Belandia B. Orford R. L. Hurst H. C. Parker M. G. (2002). Targeting of SWI/SNF chromatin remodelling complexes to estrogen-responsive genes. *EMBO J.* 21 4094–4103. 10.1093/emboj/cdf412 12145209 PMC126156

[B6] Berry N. B. Fan M. Nephew K. P. (2008). Estrogen receptor-α hinge-region lysines 302 and 303 regulate receptor degradation by the proteasome. *Mol. Endocrinol.* 22 1535–1551. 10.1210/me.2007-0449 18388150 PMC2453605

[B7] Bokhman J. V. (1983). Two pathogenetic types of endometrial carcinoma. *Gynecol. Oncol.* 15 10–17. 10.1016/0090-8258(83)90111-7 6822361

[B8] Bosse T. Haar N. T. Seeber L. M. Diest P. J. Hes F. J. Vasen H. F. A. (2013). Loss of ARID1A expression and its relationship with PI3K-Akt pathway alterations, TP53 and microsatellite instability in endometrial cancer. *Mod. Pathol.* 26 1525–1535. 10.1038/modpathol.2013.96 23702729

[B9] Carlson M. J. Thiel K. W. Yang S. Leslie K. K. (2012). Catch it before it kills: progesterone, obesity, and the prevention of endometrial cancer. *Discov. Med.* 14 215–222. 10.1016/j.biotechadv.2011.08.021.Secreted 23021376 PMC3964851

[B10] Cerami E. Gao J. Dogrusoz U. Gross B. E. Sumer S. O. Aksoy B. A. (2012). The cBio cancer genomics portal: an open platform for exploring multidimensional cancer genomics data. *Cancer Discov.* 2 401–404. 10.1158/2159-8290.CD-12-0095 22588877 PMC3956037

[B11] Chang M. T. Asthana S. Gao S. P. Lee B. H. Chapman J. S. Kandoth C. (2015). Identifying recurrent mutations in cancer reveals widespread lineage diversity and mutational specificity. *Nat. Biotechnol.* 34 155–163. 10.1038/nbt.3391 26619011 PMC4744099

[B12] Chang Y. S. Huang H. Da Yeh K. T. Chang J. G. (2017). Identification of novel mutations in endometrial cancer patients by whole-exome sequencing. *Int. J. Oncol.* 50 1778–1784. 10.3892/ijo.2017.3919 28339086

[B13] Djordjevic B. Hennessy B. T. Li J. Barkoh B. A. Luthra R. Mills G. B. (2012). Clinical assessment of PTEN loss in endometrial carcinoma: immunohistochemistry outperforms gene sequencing. *Mod. Pathol.* 25 699–708. 10.1038/modpathol.2011.208 22301702 PMC3341518

[B14] Doll M. Abal M. Monge M. Gonzalez S. Demajo E. Colás M. (2008). Novel molecular profiles of endometrial cancer-new light through old windows. *J. Steroid Biochem. Mol. Biol.* 108 221–229. 10.1016/j.jsbmb.2007.09.020 18061438

[B15] Duska L. R. Garrett A. Rueda B. R. Haas J. Chang Y. Fuller A. F. (2001). Endometrial cancer in women 40 years old or younger. *Gynecol. Oncol.* 83 388–393. 10.1006/gyno.2001.6434 11606102

[B16] Emons G. Günthert A. Thiel F. C. Camara O. Strauss H. G. Breitbach G. P. (2013). Phase II study of fulvestrant 250 mg/month in patients with recurrent or metastatic endometrial cancer: a study of the Arbeitsgemeinschaft Gynäkologische Onkologie. *Gynecol. Oncol.* 129 495–499. 10.1016/j.ygyno.2013.02.039 23500091

[B17] Fayard E. Xue G. Parcellier A. Bozulic L. Hemmings B. A. (2010). Protein kinase B (PKB/Akt), a key mediator of the PI3K signaling pathway. *Curr. Top. Microbiol. Immunol.* 346 31–56. 10.1007/82-2010-58 20517722

[B18] Feng Q. Zhang Z. Shea M. J. Creighton C. J. Coarfa C. Hilsenbeck S. G. (2014). An epigenomic approach to therapy for tamoxifen-resistant breast cancer. *Cell Res.* 24 809–819. 10.1038/cr.2014.71 24874954 PMC4085766

[B19] Ferlay J. Soerjomataram I. Dikshit R. Eser S. Mathers C. Rebelo M. (2015). Cancer incidence and mortality worldwide: Sources, methods and major patterns in GLOBOCAN 2012. *Int. J. Cancer* 136 E359–E386. 10.1002/ijc.29210 25220842

[B20] Ferlay J. Soerjomataram I. Ervik M. Dikshit R. Eser S. Mathers C. (2013). *GLOBOCAN 2012 v1.0 Cancer Incidence and Mortality Worldwide: IARC CancerBase. No. 11 [Internet].* Available at: http://globocan.iarc.fr

[B21] Forbes S. A. Beare D. Boutselakis H. Bamford S. Bindal N. Tate J. (2017). COSMIC: Somatic cancer genetics at high-resolution. *Nucleic Acids Res.* 45 D777–D783. 10.1093/nar/gkw1121 27899578 PMC5210583

[B22] Gao J. Aksoy B. A. Dogrusoz U. Dresdner G. Gross B. Sumer S. O. (2013). Integrative analysis of complex cancer genomics and clinical profiles using the cBioPortal. *Sci. Signal.* 6:pl1. 10.1126/scisignal.2004088 23550210 PMC4160307

[B23] García-Carpizo V. Sarmentero J. Han B. Graña O. Ruiz-Llorente S. Pisano D. G. (2016). NSD2 contributes to oncogenic RAS-driven transcription in lung cancer cells through long-range epigenetic activation. *Sci. Rep.* 6:32952. 10.1038/srep32952 27604143 PMC5015087

[B24] Garg K. Soslow R. A. (2014). Endometrial carcinoma in women aged 40 years and younger. *Arch. Pathol. Lab. Med.* 138 335–342. 10.5858/arpa.2012-0654-RA 24576029

[B25] Girault I. Bièche I. Lidereau R. (2006). Role of estrogen receptor α transcriptional coregulators in tamoxifen resistance in breast cancer. *Maturitas* 54 342–351. 10.1016/j.maturitas.2006.06.003 16822624

[B26] Giudice F. S. Squarize C. H. (2013). The determinants of head and neck cancer: unmasking the PI3K pathway mutations. *J. Carcinog. Mutagen.* Suppl 5 432–435. 10.4172/2157-2518.S5-003 25126449 PMC4130654

[B27] Guan B. Gao M. Wu C.-H. Wang T.-L. Shih I.-M. (2012). Functional analysis of In-frame Indel ARID1A mutations reveals new regulatory mechanisms of its tumor suppressor functions. *Neoplasia* 14 986–993. 10.1593/neo.121218 23097632 PMC3479842

[B28] Han S. Y. Kato H. Kato S. Suzuki T. Shibata H. Ishii S. (2000). Functional evaluation of PTEN missense mutations using in vitro phosphoinositide phosphatase assay. *Cancer Res.* 60 3147–3151. 10866302

[B29] Huang Z. Wu H. Chuai S. Xu F. Yan F. Englund N. (2013). NSD2 Is recruited through Its PHD domain to oncogenic gene loci to drive multiple myeloma. *Cancer Res.* 73 6277–6288. 10.1158/0008-5472.CAN-13-1000 23980095

[B30] Huether R. Dong L. Chen X. Wu G. Parker M. Wei L. (2014). The landscape of somatic mutations in epigenetic regulators across 1000 pediatric cancer genomes. *Nat. Commun.* 5:3630. 24710217 10.1038/ncomms4630 PMC4119022

[B31] Jaffe J. D. Wang Y. Chan H. M. Zhang J. Huether R. Kryukov G. V. (2013). Global chromatin profiling reveals NSD2 mutations in pediatric acute lymphoblastic leukemia. *Nat. Genet.* 45 1386–1393. 10.1038/ng.2777 24076604 PMC4262138

[B32] Jaiswal B. S. Kljavin N. M. Stawiski E. W. Chan E. Parikh C. Durinck S. (2013). Oncogenic ERBB3 mutations in human cancers. *Cancer Cell* 23 603–617. 10.1016/j.ccr.2013.04.012 23680147

[B33] Jeong E. G. Soung Y. H. Woo L. J. Hak L. S. Woo N. S. Lee J. Y. (2006). ERBB3 kinase domain mutations are rare in lung, breast and colon carcinomas. *Int. J. Cancer* 119 2986–2987. 10.1002/ijc.22257 16998794

[B34] Jones S. Li M. Williams Parsons D. Zhang X. Wesseling J. Kristel P. (2012). Somatic mutations in the chromatin remodeling gene ARID1A occur in several tumor types. *Hum. Mutat.* 33 100–103. 10.1002/humu.21633 22009941 PMC3240719

[B35] Kanehisa M. Sato Y. Kawashima M. Furumichi M. Tanabe M. (2016). KEGG as a reference resource for gene and protein annotation. *Nucleic Acids Res.* 44 D457–D462. 10.1093/nar/gkv1070 26476454 PMC4702792

[B36] Kang H. B. Choi Y. Lee J. M. Choi K. C. Kim H. C. Yoo J. Y. (2009). The histone methyltransferase, NSD2 enhances androgen receptor-mediated transcription. *FEBS Lett.* 583 1880–1886. 10.1016/j.febslet.2009.05.038 19481544

[B37] Kuhn E. Wu R. C. Guan B. Wu G. Zhang J. Wang Y. (2012). Identification of molecular pathway aberrations in uterine serous carcinoma by genome-wide analyses. *J. Natl. Cancer Inst.* 104 1503–1513. 10.1093/jnci/djs345 22923510 PMC3692380

[B38] Lax S. F. Kendall B. Tashiro H. Slebos R. J. Hedrick L. (2000). The frequency of p53 K- mutations, and microsatellite instability differs in uterine ras evidence endometrioid and serous carcinoma of distinct molecular genetic pathways. *Cancer* 88 814–824. 10.1002/(SICI)1097-0142(20000215)88:4<814::AID-CNCR12>3.0.CO;2-U 10679651

[B39] Le Gallo M. O’Hara A. J. Rudd M. L. Urick M. E. Hansen N. F. O’Neil N. J. (2012). Exome sequencing of serous endometrial tumors identifies recurrent somatic mutations in chromatin-remodeling and ubiquitin ligase complex genes. *Nat. Genet.* 44 1310–1315. 10.1038/ng.2455 23104009 PMC3515204

[B40] Li J. (1997). PTEN, a putative protein tyrosine phosphatase gene mutated in human brain, breast, and prostate cancer. *Science* 275 1943–1947. 10.1126/science.275.5308.1943 9072974

[B41] Li M. Zhang Z. Li X. Ye J. Wu X. Tan Z. (2014). Whole-exome and targeted gene sequencing of gallbladder carcinoma identifies recurrent mutations in the ErbB pathway. *Nat. Genet.* 46 872–876. 10.1038/ng.3030 24997986

[B42] Li Y. Trojer P. Xu C. F. Cheung P. Kuo A. Drury W. J. (2009). The target of the NSD family of histone lysine methyltransferases depends on the nature of the substrate. *J. Biol. Chem.* 284 34283–34295. 10.1074/jbc.M109.034462 19808676 PMC2797197

[B43] Liang H. Cheung L. W. T. Li J. Ju Z. Yu S. Stemke-Hale K. (2012). Whole-exome sequencing combined with functional genomics reveals novel candidate driver cancer genes in endometrial cancer. *Genome Res.* 22 2120–2129. 10.1101/gr.137596.112 23028188 PMC3483541

[B44] Livak K. J. Schmittgen T. D. (2001). Analysis of relative gene expression data using real-time quantitative PCR and the 2-ΔΔCT method. *Methods* 25 402–408. 10.1006/meth.2001.1262 11846609

[B45] Lu C. Allis C. D. (2017). SWI/SNF complex in cancer. *Nat. Genet.* 49 178–179. 10.1038/ng.3779 28138149 PMC5617137

[B46] Maehama T. Dixon J. E. (1998). The tumor suppressor, PTEN/ MMAC1 dephosphorylates the lipid second messenger, phosphatidylinositol 345-trisphosphate. *J. Biol. Chem.* 273 13375–13379. 10.1074/jbc.273.22.13375 9593664

[B47] Maniketh I. Ravikumar G. Crasta J. A. Prabhu R. Vallikad E. (2014). Estrogen and progesterone receptor expression in endometrioid endometrial carcinomas: a clinicopathological study. *Middle East J. Cancer* 5 67–73.

[B48] Morice P. Leary A. Creutzberg C. Abu-Rustum N. Darai E. (2016). Endometrial cancer. *Gynecol. Obstet. Fertil.* 44 239–243. 10.1016/S0140-6736(15)00130-0 27053036

[B49] Nadji M. Gomez-Fernandez C. Ganjei-Azar P. Morales A. R. (2005). Immunohistochemistry of estrogen and progesterone receptors reconsidered: experience with 5993 breast cancers. *Am. J. Clin. Pathol.* 123 21–27. 10.1309/4WV7-9N2G-HJ3X-1841 15762276

[B50] Naguib A. Bencze G. Cho H. Zheng W. Tocilj A. Elkayam E. (2015). PTEN functions by recruitment to cytoplasmic vesicles. *Mol. Cell* 58 255–268. 10.1016/j.molcel.2015.03.011 25866245 PMC4423730

[B51] O’Hara A. J. Bell D. W. (2012). The genomics and genetics of endometrial cancer. *Adv. Genomics Genet.* 2012 33–47. 10.2147/AGG.S28953 22888282 PMC3415201

[B52] Olayioye M. A. Neve R. M. Lane H. A. Hynes N. E. (2000). The ErbB signaling network: receptor heterodimerization in development and cancer. *EMBO J.* 19 3159–3167. 10.1093/emboj/19.13.3159 10880430 PMC313958

[B53] Pecorelli S. (2009). FIGO committee on gynecology oncology revised FIGO staging for carcinoma of the vulva, cervix, and endometrium. *Int. J. Gynecol. Obstet.* 105 103–104. 10.1016/j.ijgo.2009.02.012 19367689

[B54] Prasad M. Wang H. Douglas W. Barakat R. R. Ellenson L. H. (2005). Molecular genetic characterization of tamoxifen-associated endometrial cancer. *Gynecol. Oncol.* 96 25–31. 10.1016/j.ygyno.2004.08.046 15589576

[B55] Prat J. Gallardo A. Cuatrecasas M. Catasús L. (2007). Endometrial carcinoma: pathology and genetics. *Pathology* 39 72–87. 10.1080/00313020601136153 17365824

[B56] Rao B. Shibata Y. Strahl B. D. Lieb J. D. (2005). Dimethylation of histone H3 at Lysine 36 demarcates regulatory and nonregulatory chromatin genome-wide. *Mol. Cell Biol.* 25 9447–9459. 10.1128/MCB.25.21.9447-9459.2005 16227595 PMC1265832

[B57] Sithanandam G. Anderson L. M. (2008). The ERBB3 receptor in cancer and cancer gene therapy. *Cancer Gene Ther.* 15 413–448. 10.1038/cgt.2008.15 18404164 PMC2761714

[B58] Talhouk A. McAlpine J. N. (2016). New classification of endometrial cancers: the development and potential applications of genomic-based classification in research and clinical care. *Gynecol. Oncol. Res. Pract.* 3:14. 10.1186/s40661-016-0035-4 27999680 PMC5154099

[B59] Tamborero D. Rubio-Perez C. Deu-Pons J. Schroeder M. P. Vivancos A. Rovira A. (2018). Cancer genome interpreter annotates the biological and clinical relevance of tumor alterations. *Genome Med.* 10:25. 10.1186/s13073-018-0531-8 29592813 PMC5875005

[B60] Temkin S. M. Fleming G. (2009). Current treatment of metastatic endometrial cancer. *Cancer Control* 16 38–45. 10.1177/107327480901600106 19078928

[B61] The Cancer Genome Atlas Network (2012). Comprehensive molecular portraits of human breast tumours. *Nature* 490 61–70. 10.1038/nature11412 23000897 PMC3465532

[B62] The Cancer Genome Atlas Research Network Kandoth C. Schultz N. Cherniack A. D. Akbani R. Liu Y. (2013). Integrated genomic characterization of endometrial carcinoma. *Nature* 497 67–73. 10.1038/nature12113 23636398 PMC3704730

[B63] Thorvaldsdóttir H. Robinson J. T. Mesirov J. P. (2013). Integrative Genomics Viewer (IGV): high-performance genomics data visualization and exploration. *Brief. Bioinform.* 14 178–192. 10.1093/bib/bbs017 22517427 PMC3603213

[B64] Toyokawa G. Cho H. S. Masuda K. Yamane Y. Yoshimatsu M. Hayami S. (2011). Histone lysine methyltransferase Wolf-hirschhorn syndrome candidate 1 is involved in human carcinogenesis through regulation of the Wnt pathway. *Neoplasia* 13 887–898. 10.1593/neo.11048 22028615 PMC3201566

[B65] Van Allen E. M. Wagle N. Levy M. A. (2013). Clinical analysis and interpretation of cancer genome data. *J. Clin. Oncol.* 31 1825–1833. 10.1200/JCO.2013.48.7215 23589549 PMC4878102

[B66] Wang K. Li M. Hakonarson H. (2010). ANNOVAR: functional annotation of genetic variants from high-throughput sequencing data. *Nucleic Acids Res.* 38:e164. 10.1093/nar/gkq603 20601685 PMC2938201

[B67] Wilson B. G. Roberts C. W. M. (2011). SWI/SNF nucleosome remodellers and cancer. *Nat. Rev. Cancer* 11 481–492. 10.1038/nrc3068 21654818

[B68] Xu J. Li Z. Wang J. Chen H. Fang J. Y. (2014). Combined PTEN mutation and protein expression associate with overall and disease-free survival of glioblastoma patients. *Transl. Oncol.* 7 196–205. 10.1016/j.tranon.2014.02.004 24721394 PMC4101389

[B69] Zannoni G. F. Monterossi G. De Stefano I. Gargini A. Salerno M. G. Farulla I. (2013). The expression ratios of estrogen receptor alpha (ERalpha) to estrogen receptor beta1 (ERbeta1) and ERalpha to ERbeta2 identify poor clinical outcome in endometrioid endometrial cancer. *Hum. Pathol.* 44 1047–1054. 10.1016/j.humpath.2012.09.007 23266443

[B70] Zhang J. Baran J. Cros A. Guberman J. M. Haider S. Hsu J. (2011). International cancer genome consortium data portal-a one-stop shop for cancer genomics data. *Database* 2011:bar026. 10.1093/database/bar026 21930502 PMC3263593

[B71] Zhang X. Zhang Y. Yang Y. Niu M. Sun S. Ji H. (2012). Frequent low expression of chromatin remodeling gene ARID1A in breast cancer and its clinical significance. *Cancer Epidemiol.* 36 288–293. 10.1016/j.canep.2011.07.006 21889920

