# Supplementary figures and images for: Targeted Next-Generation Sequencing Identifies Actionable Targets in Estrogen Receptor Positive and Estrogen Receptor Negative Endometriod Endometrial Cancer
Source: Front Pharmacol. 2018 Jul 13;9:750. doi: 10.3389/fphar.2018.00750 (PMC6053487; doi:10.3389/fphar.2018.00750)

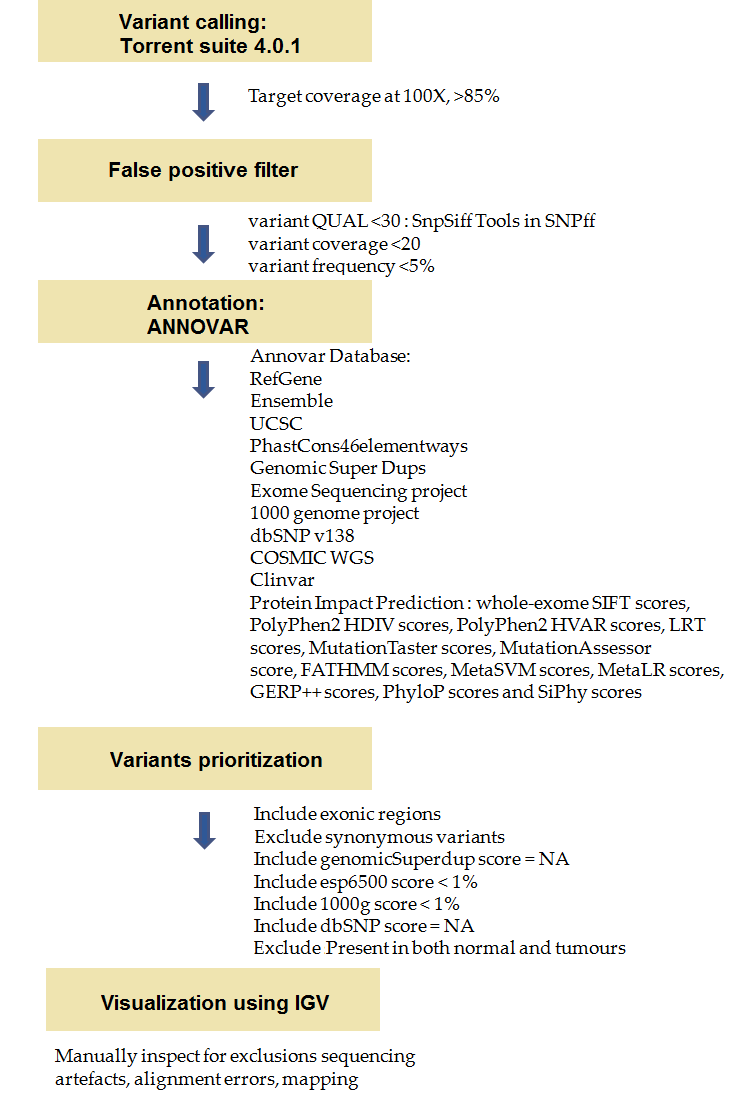

Supplement: FIGURE S1 — Variant analysis and prioritization workflow. Summary of our variant evaluation process for identifying candidate mutations. [file Image_1.tif]

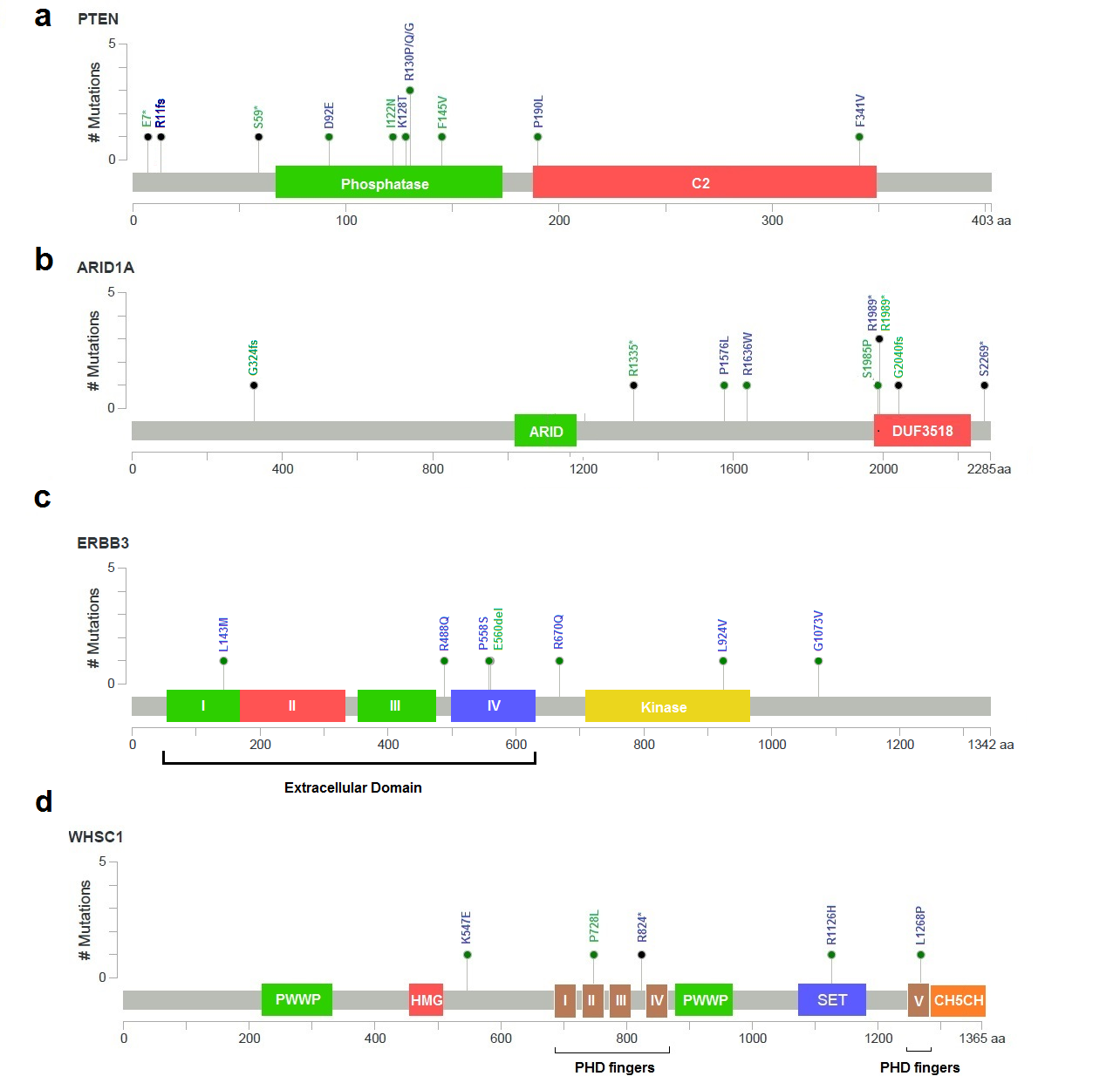

Supplement: FIGURE S2 — Mutations distribution across exon and functional domain of (a) PTEN, (b) ARID1A, (c) ERBB3, and (d) WHSC1. Any position with a mutation obtains a circle; the length of the line depends on the number of mutations detected at that codon. The gray bar represents the entire protein with the different amino acid positions (aa). The colored boxes are specific functional domains. On top of the lollipops variants are annotated as the amino-acid change at that specific site. Blue letter indicates mutation occur in ER positive subtype while green letter indicate mutation occur in ER negative subtype of our analysis. [file Image_2.tif]
